# Bud endodormancy in deciduous fruit trees: advances and prospects

**DOI:** 10.1038/s41438-021-00575-2

**Published:** 2021-06-01

**Authors:** Qinsong Yang, Yuhao Gao, Xinyue Wu, Takaya Moriguchi, Songling Bai, Yuanwen Teng

**Affiliations:** 1 College of Agriculture and Biotechnology, Zhejiang University, Hangzhou Zhejiang, 310058 China; 2 Key Laboratory for Silviculture and Conservation, Ministry of Education, Beijing Forestry University, Haidian District, Beijing, 100083 China; 3 Shizuoka Professional University of Agriculture, Iwata Shizuoka, 438-0803 Japan; 4 Hainan Institute of Zhejiang University, Sanya, Hainan 572000 China

**Keywords:** Plant molecular biology, Plant signalling

## Abstract

Bud endodormancy is a complex physiological process that is indispensable for the survival, growth, and development of deciduous perennial plants. The timely release of endodormancy is essential for flowering and fruit production of deciduous fruit trees. A better understanding of the mechanism of endodormancy will be of great help in the artificial regulation of endodormancy to cope with climate change and in creating new cultivars with different chilling requirements. Studies in poplar have clarified the mechanism of vegetative bud endodormancy, but the endodormancy of floral buds in fruit trees needs further study. In this review, we focus on the molecular regulation of endodormancy induction, maintenance and release in floral buds of deciduous fruit trees. We also describe recent advances in quantitative trait loci analysis of chilling requirements in fruit trees. We discuss phytohormones, epigenetic regulation, and the detailed molecular network controlling endodormancy, centered on *SHORT VEGETATIVE PHASE* (*SVP*) and *Dormancy-associated MADS-box* (*DAM*) genes during endodormancy maintenance and release. Combining previous studies and our observations, we propose a regulatory model for bud endodormancy and offer some perspectives for the future.

## Introduction

Dormancy refers to a temporary suspension of visible growth of any plant structure containing a meristem^1^ and is a biological characteristic of higher plants adapted to seasonal environmental changes through long-term natural selection^2^. Bud dormancy is an important physiological process that helps plants survive harsh winter weather and determines growth resumption and flowering in the following spring. Bud dormancy is generally divided into three types: paradormancy, endodormancy, and ecodormancy according to various factors that cause dormancy^1^. This classification has been widely accepted by researchers in the study of bud dormancy. In this review, we mainly discuss endodormancy.

**Fig. 1:**
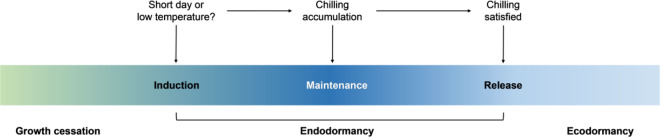
Dormancy–growth cycle. After growth cessation and bud set, low temperatures and/or the short-day photoperiod induces trees to enter endodormancy. When the chilling requirement is satisfied, endodormancy is released and trees enter the ecodormancy stage. Endodormancy is divided into three stages: induction (establishment), maintenance, and release

In autumn, with decreased temperature and/or day length, buds (both terminal buds and axillary buds) are induced to enter endodormancy. In this stage, the buds cannot sprout, even under favorable conditions. After the fulfillment of a certain period of chilling accumulation (termed chilling requirement, CR), endodormancy is released, and buds acquire the potential capability to resume growth^3,4^. Because the release of endodormancy generally occurs at the depth of winter, buds cannot break immediately because of unfavorable conditions and are forced into ecodormancy (Fig. 1). With increasing temperature, bud growth resumes in the spring. According to Howe et al.^5^ and Singh et al.^6^, endodormancy is further divided into three stages: induction (establishment), maintenance, and release (Fig. 1). Endodormancy maintenance refers to the process from the beginning of chilling accumulation until endodormancy release^5^. In this stage, the buds could not burst unless chilling accumulation was satisfied. With prolonged chilling accumulation, the factors required for endodormancy maintenance are eliminated, and endodormancy is released.

**Fig. 2:**
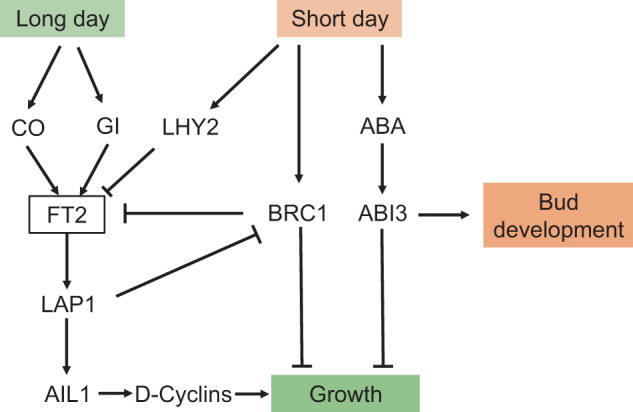
A molecular model for growth cessation based on studies in poplar. FT2 acts as a central regulator of active growth. Downregulation of *FT2* causes growth cessation. Under long-day conditions, *FT2* has a high expression level under the control of CO, GI, and other genes, thus promoting *LAP1* and *AIL1* expression and maintaining tree growth. When autumn approaches, the photoperiod is shortened, which promotes the expression of *LHY2* and *BRC1* and suppresses *FT2* so that growth is inhibited. In addition, the ABA content increases, promoting *ABI3* expression and bud development^16^. Arrowheads denote positive effects; blocked arrows denote negative effects

During the past decade, significant progress has been made in studying the mechanism of growth cessation and bud endodormancy in various species, especially in the model plant poplar (*Populus* spp.). Growth cessation is a necessary process prior to bud endodormancy. With the shortened day length in autumn, poplar ceases growth and forms apical buds containing leaf primordia and shoot apical meristems. Böhlenius et al.^7^ found that two genes, *CONSTANS* (*CO*) and *FLOWERING LOCUS T* (*FT*), which normally regulate flowering time, also regulate the induction of growth cessation and bud set under short days. *FT2* is expressed in the leaves, and the FT2 protein is translocated to the apex, where it promotes growth; downregulation of *FT2* in leaves results in reduced levels of the FT2 protein in the apex^8^. Genes related to the circadian clock also participate in growth cessation. Ding et al.^9^ showed that the circadian clock gene *GIGANTEA* (*GI*) directly regulates the expression of *FT2* independent of CO, thereby regulating short-day-induced growth cessation and apical bud formation in poplar. Downregulation of *LHY* and *TOC1* delays growth cessation and bud set^10^, and *LHY2* is necessary and sufficient to cause *FT2* downregulation in a night-length-dependent manner^11^. FT2 downregulation results in inhibition of the expression of the downstream components *LAP1* (the homologous gene of *APETALA1*, a characteristic gene of Arabidopsis floral meristem) and AIL1 (the homologous gene of *AINTEGUMENTA* in Arabidopsis), which in turn eventually leads to growth cessation and bud formation^12–14^. Recent studies have shown that in addition to the CO/FT pathway, BRC1 (homologous to Arabidopsis gene *BRANCHED 1*) can form a feedback loop controlling seasonal growth in response to short days^15^ (Fig. 2). In addition, the content of the plant hormone abscisic acid (ABA) increases under short days, promoting *ABI3* expression and bud development^16^.

After growth cessation, buds enter endodormancy in response to the autumn environment. Abscisic acid (ABA) is centrally important in establishing bud endodormancy^17^. Studies on poplar indicate a clear network of endodormancy regulation. ABA mediates callose deposition and plasmodesmata closure and blocks the transport of growth-promoting substances such as FT1 to shoot meristems, thus leading the buds to endodormancy; however, ABA does not affect growth cessation^17^. This result further proves that growth cessation and bud endodormancy are two different processes^17^. After prolonged chilling accumulation, GA biosynthesis genes (e.g., *GA20ox8* and *GA3ox1*) are upregulated, and callose is degraded, allowing FT to be transported to shoot meristems, where it induces endodormancy release and bud break^17,18^. In poplar, the MADS-box gene *SVL* (*SHORT VEGATATIVE PHASE LIKE*), which is induced by ABA, is a crucial transcription factor promoting bud endodormancy. SVL mediates short-photoperiod- and low-temperature-induced terminal bud endodormancy and directly promotes the expression of the ABA synthesis gene *NCED3*, forming a positive feedback regulation with ABA signaling^17,19^. In addition, SVL promotes the expression of *CALS1*, a callose synthetase that promotes callose deposition, resulting in the closure of plasmodesmata, thus blocking intercellular communication. At the same time, SVL also inhibits the expression of *GA20ox1* and *GA20ox2* and promotes the expression of *GA2ox8*, thus reducing the content of gibberellin (GA) and deepening endodormancy^19,20^. Therefore, SVP/SVL can integrate the metabolism of ABA and GA, regulating the maintenance of endodormancy. Reducing the GA content by overexpressing the GA catabolism gene *GA2ox* also promotes apical bud formation and delays endodormancy release^19,21^. EARLY BUD BREAK 1 (EBB1), which suppresses *SVL* expression, is positively regulated by low temperature, leading to the upregulation of *EBB3*. EBB3 directly activates *CYCD3.1*, promoting cell division and bud break^22^. In addition to transcriptional regulation, epigenetic regulation, including DNA methylation, is also reported to be involved in growth cessation and bud endodormancy processes in poplar. The DNA demethylase DEMETER-LIKE is chilling responsive and mediates poplar bud formation and bud break^23,24^.

Although these findings in the model plant poplar help us greatly understand the biology of the bud dormancy cycle, most of the studies have focused on vegetative buds. However, in the case of fruit trees, the dormancy of floral buds should be given more attention because it is a critical component in fruit production. In this review, we focus on bud endodormancy of deciduous fruit trees, summarizing the recent progress on endodormancy regulation and discussing the scientific questions still to be addressed to further understand the mechanism of bud endodormancy.

## Endodormancy induction in deciduous fruit trees

In many deciduous fruit trees, such as pear and apple, growth cessation and flower bud differentiation often occur in early summer (May–June in China)^25^ when the days become longer and the temperature higher^25^, while the establishment of endodormancy happens in late autumn when the days become shorter and the temperature lower. Hence, the growth cessation of shoots in some deciduous fruit trees is not strictly caused by short-day conditions. Heide and Prestrud^26^ showed that growth arrest and bud formation in pear and apple could be induced by artificial low temperatures rather than short days. However, under natural conditions, growth cessation occurs in summer at relatively high temperatures. Therefore, the factors regulating the growth cessation of deciduous fruit trees under natural conditions need further study, as Cooke et al.^27^ also commented.

**Fig. 3:**
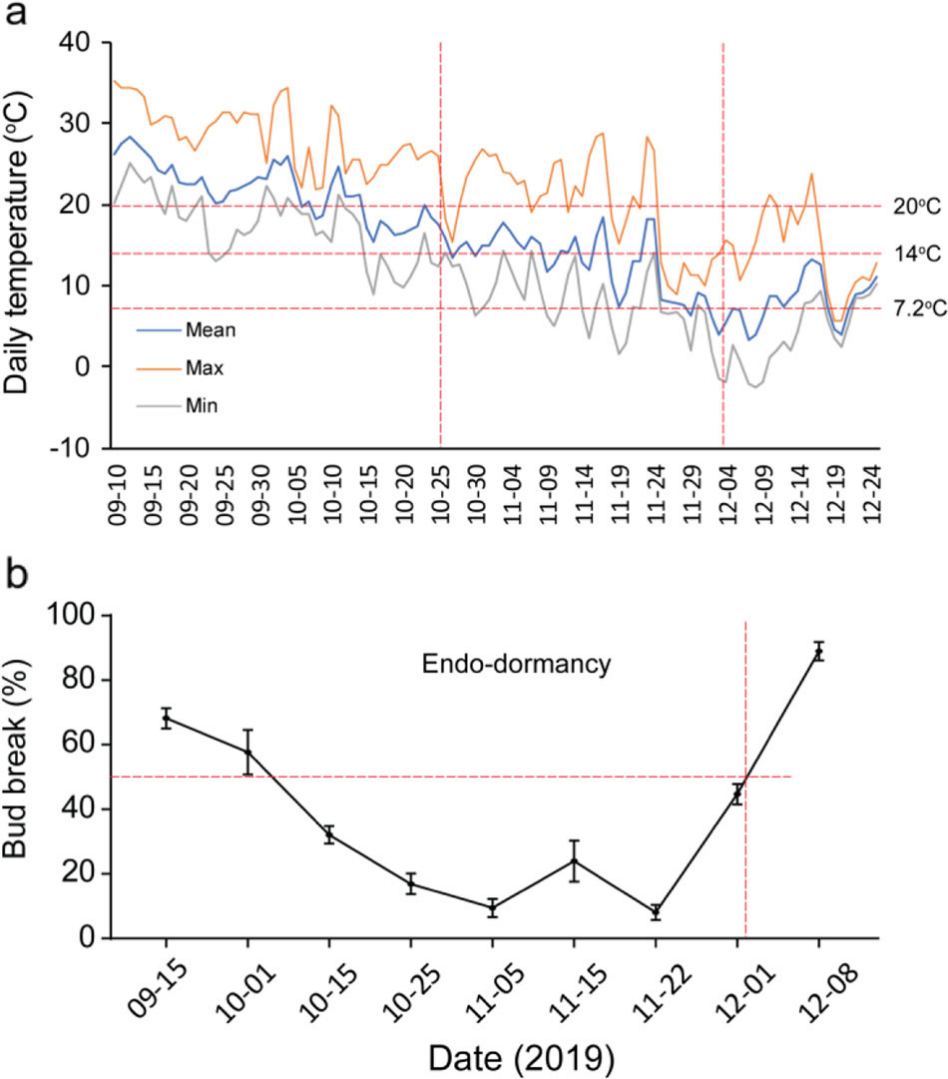
Daily temperature and bud break percentage of ‘Cuiguan' pear in Hangzhou. Daily mean temperature in Hangzhou (**a**) and bud-break percentage indicating the dormancy status of ‘Cuiguan’ pear (**b**) in autumn and winter of 2019. The endodormancy stage is indicated between the vertical dashed red lines. Horizontal dashed red lines indicate temperatures as shown (**a**) and 50% bud breakage (**b**). Branches were sampled at the time points shown and were kept in floral foam in forcing conditions for 21 days for measuring the bud-break percentage. When the trees entered endodormancy (start of the period with the lowest bud-break percentage), the daily mean temperature was still ~14 °C, suggesting that the critical temperature to induce endodormancy of ‘Cuiguan’ pear is possibly not lower than 14 °C. When dormancy was released, the temperature was just approximately 7.2 °C, suggesting that the 7.2 °C model might not be suitable for calculating the chilling requirement of ‘Cuiguan’ pear in the Hangzhou area

Induction of bud endodormancy often occurs in autumn. In peach, bud endodormancy is induced by both short-day and low-temperature conditions^28^, while in grape, bud endodormancy was shown to be induced by short-day length or low temperature in different grape genotypes^29^. Heide and Prestrud^26^ concluded that endodormancy of European pear and apple could be induced by low temperature (<12 °C) rather than a short-day length under artificial conditions. However, growth cessation and bud endodormancy were not clearly distinguished in their study. It is difficult to determine whether plants in their study were in the endodormancy stage or had just ceased growth, because even if the trees ceased growth and formed apical buds, the apical buds might sprout when early defoliation occurs in summer^30^. According to our observations, under natural conditions in Hangzhou, China (30° 06′ N; 119° 55′ E), the bud-break percentage of ‘Cuiguan’ pear (*Pyrus pyrifolia*) under forcing conditions decreased from September and reached its lowest in late October. During this period, the daily mean temperature decreased from c. 25 °C to c. 14 °C, much higher than the temperature used in Heide and Prestrud’s experiments^26^ (Fig. 3). A similar phenomenon was observed in another pear cultivar, ‘Suli’ (*P. pyrifolia* White Pear Group), in Dangshan County, China (34° 28′ N; 116° 29′ E)^31^. It is generally believed that there is a critical day length or temperature for each species or even ecotype to induce bud endodormancy. However, for most fruit trees, the critical temperature/day length for endodormancy induction still needs further study. In addition to a low temperature and short photoperiod changes in other environmental cues (e.g., air humidity or soil moisture content) might also be included as putative endodormancy inducers. Therefore, the environmental signals for bud endodormancy induction are still obscure in most fruit tree species and may differ with the local environment.

In addition to environmental inducers, exogenous ABA treatments have been found to promote the establishment of bud endodormancy in deciduous fruit trees^31–33^. During the endodormancy induction stage of grape buds, endogenous ABA increased^34^. In grape, overexpression of *CYP707A4*, a gene encoding an ABA 8′‐hydroxylase caused a lower ABA content and increased lateral bud out-growth and shoot length^35^, suggesting that ABA might be the main regulator of growth cessation in grape. However, the effects of ABA on endodormancy induction have not been determined^35^. In our observations over several years, the ABA content in pear buds peaked during the endodormancy maintenance stage rather than the induction stage^31,33^. Therefore, whether bud endodormancy of deciduous fruit trees is induced by endogenous ABA under natural conditions remains unclear because of the lack of a genetic analysis approach.

## Endodormancy maintenance and release in deciduous fruit trees

The maintenance and release of bud endodormancy are consecutive processes. After the induction of endodormancy, with increasing chilling hours, the factors that maintain bud endodormancy are gradually inhibited, and bud endodormancy is eventually released. Whether the endodormancy of buds can be released on time to enable commercial fruit production is a matter directly related to bud burst and flowering time and therefore affects yield and fruit quality.

### Chilling requirement

The chilling requirement (CR) is directly related to endodormancy maintenance and release and closely related to flowering time. Therefore, an accurate evaluation of the CR is important, and various CR models have been developed. Weinberger^36^ demonstrated that temperatures below 45 °F (~7.2 °C) are effective for breaking peach endodormancy. Although a 7.2 °C model has been used for many years, there are some problems because different fruit trees may have their own specific low temperature. Thus, Richardson et al.^37^ proposed a Utah model, demonstrating that different extents of chilling have different effects on endodormancy. The 7.2 °C model and Utah model are widely used at present, while the 0–7.2 °C model, 0–14 °C model, positive Utah model, and modified Utah model, which are all modified from the 7.2 °C and Utah models, are suitable for special areas or climates^38^. The CR differs among plant species and cultivars and is also affected by climate and other environmental factors. For example, ‘Suli’ pear floral buds need approximately 800–1000 hours of chilling^39,40^, while ‘Cuiguan’ pear floral buds only need approximately 200–400 chilling hours to break endodormancy^39^. However, it is often difficult to determine the start time of chilling accumulation, resulting in inaccurate CR determination. In our recent observations, endodormancy of ‘Cuiguan’ pear was released when the daily mean temperature was above 7.2 °C in Hangzhou, China (Fig. 3), while the Utah model indicated that the CR was 113 chilling units (CU), much lower than that in previous studies (c. 300 CU)^39,41^. This phenomenon suggests that CR models are not universal for different conditions, and CR data acquired under natural conditions might still need to be modified by applying artificial chilling accumulation at a fixed temperature. As 4–5 °C is efficient for the endodormancy release of many trees, researchers often use that temperature to break endodormancy^18,40,42^. Thus, we can calculate the CRs more accurately by using artificial chilling accumulation.

Obviously, CR is a typical quantitative trait and therefore amenable to study by QTL analysis. Fan et al.^43^ located CR-related QTLs in peach on linkage groups G1, G4, G5, G6, G7, and G8 using a population of 378 F2 individuals. Interestingly, the major QTLs in G1 were also associated with flowering time. Rowland et al.^44^ identified two CR-associated QTLs in blueberry from a genetic linkage map using simple repeat sequence (SSR) markers. Castède et al.^45^ identified dormancy- and flowering-related QTLs and screened 57 out of 79 candidate genes for colocalization with these QTLs. Among them, *LHY*, which was reported to regulate growth cessation and bud break in poplar, was located on LG2 in the CR-related QTL but not in the QTL related to flowering time and heat requirements. *HYL1* of sweet cherry was located on LG1 together with *DAM5* and *DAM6* in the QTL related to flowering time, which was consistent with findings in peach, showing that this genomic region is conserved between sweet cherry and peach^45^. Recently, QTLs related to the downregulation of *PmDAM6*, endodormancy release, and bud burst in Japanese apricot, were identified, further demonstrating that the downregulation of *DAM* genes is involved in bud endodormancy release and bud burst^46^. Gabay et al.^47,48^ identified QTLs associated with bud burst in European pear and found collinearity of the genetic regions controlling the CR between apple and pear. Recently, these researchers further carried out RNA-seq and metabolic profiling, combined with QTL analysis, and identified α-linolenic acid as an important metabolite in relation to the CR^49^. Although much progress has been made in CR research in different species, we are not yet able to fully explain the mechanistic basis of the CR.

### Roles of phytohormones

Phytohormones play an important role in the dormancy–growth cycle^4,16,27^. Among them, ABA and GA are the two most important hormones that antagonistically regulate bud endodormancy induction, maintenance, and release^50^. A high level of endogenous ABA is the primary factor in maintaining bud endodormancy^35^, while GA is responsible for endodormancy release^51^.

In pear, the ABA content increased during endodormancy maintenance and decreased during endodormancy release^31,52^. The decrease in ABA content during endodormancy release was related to the downregulation of ABA synthesis genes and the upregulation of genes for ABA catabolism in pear and grape buds^53–55^. Exogenous ABA treatment caused delayed endodormancy release, while treatment with fluridone, an ABA biosynthesis inhibitor, promoted endodormancy release in pear^40^, suggesting that ABA plays a crucial role in endodormancy maintenance, which is consistent with studies on poplar^19,22^. Overexpression of the ABA-responsive transcription factor PpyABF3 caused growth inhibition in pear calli with downregulation of *CYCLIN-D* and *EXPANSIN A1*^40^, indicating that ABA promotes endodormancy maintenance, possibly by inhibiting cell division and cell expansion. Recent studies on pear showed that the expression of *PpyCYP707A3*, a gene related to ABA catabolism, increased continuously and linearly during the chilling accumulation process^31,40^. At the same time, the ABA content decreased, and bud break increased^31,52^, which is similar to findings in peach^50^. In addition, overexpression of *VvCYP707A4* reduced the ABA content in grape buds, promoting endodormancy release and leading to enhanced regrowth^35^.

In contrast to ABA, GA may be related to endodormancy release. The GA content in peach buds increased with accumulated chilling hours^56^. During the process of endodormancy release, the expression of gibberellin-related genes, such as *GA3OX*, *GA20OX,* and *GASA*, was significantly upregulated, and the GA content in buds increased significantly in grape and pear^57,58^, which is similar to observations in poplar^18,59^. Exogenous GA treatments were effective in promoting endodormancy release only after a period of chilling accumulation in Japanese apricot and pear^40,51,58^. However, the mechanism of chilling-induced GA accumulation remains obscure, and how GAs function in endodormancy release in deciduous fruit trees is still not clear.

Moreover, the regulation of ABA and GA in the dormancy cycle usually occurs through their crosstalk. Our recent study showed that pear GAST1, which promotes GA biosynthesis, is inhibited by ABA^58^. When ABA is degraded with the upregulation of *CYP707A* during prolonged chilling accumulation, GAST1 is released from ABA inhibition, resulting in an increase in the GA content. ABA-responsive ABF3 in pear also induced the expression of *GA2OX1*, which might lead to a lower GA content. Thus, ABA seems to promote GA catabolism and inhibit GA biosynthesis, thus maintaining bud endodormancy in pear, which is similar to the results in poplar^19,20^. However, the transcription factors at the intersection of ABA and GA during bud endodormancy are still obscure.

In addition to GA and ABA, other phytohormones are also involved in the endodormancy process. Studies on tea plants showed that the IAA content in a terminal and lateral buds increased gradually during endodormancy release^60^, while studies on the adventitious buds of leafy spurge showed that the IAA content was higher in the paradormancy and endodormancy stages and lower in the ecodormancy stage^61^. A study in grape showed that ethylene might also participate in the process of bud endodormancy release^62^. However, there is still a lack of genetic approaches that would provide solid evidence to establish the regulatory roles of these phytohormones in bud endodormancy.

### Molecular network of DAM/SVP-centered bud endodormancy regulation

**Fig. 4:**
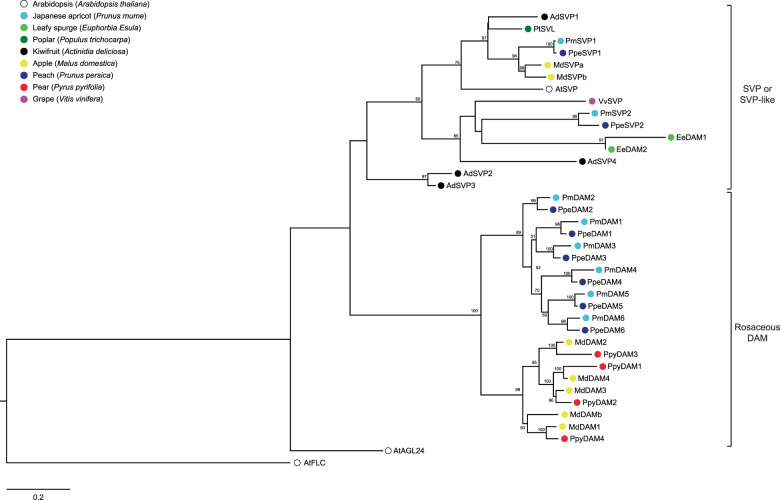
Phylogenetic analysis of DAMs/SVPs from several plant species. Proteins used in the analysis are as follows: AtAGL24 (AT4G24540), AtFLC (AT5G10140), AtSVP (AT2G22540), AdSVP1 (AFA37963), AdSVP2 (AFA37964), AdSVP3 (AFA37965), AdSVP4 (AFA37966), EeDAM1 (ABY53594), EeDAM2 (ABY60423), MdDAM1 (AOA32865), MdDAM2 (AOA32866), MdDAM3 (XP_028963037.1), MdDAM4 (AOA32868), MdDAMb (ADL36743), MdSVPa (AOA32867), MdSVPb (BAR40332), PmDAM1 (BAK78921), PmDAM2 (BAK78922), PmDAM3 (BAK78923), PmDAM4 (BAK78924), PmDAM5 (BAK78920), PmDAM6 (BAH22477), PmSVP1 (AML81015), PmSVP2 (AML81016), PpeDAM1 (ABJ96361), PpeDAM2 (ABJ96363), PpeDAM3 (ABJ96364), PpeDAM4 (ABJ96358), PpeDAM5 (ABJ96359), PpeDAM6 (ABJ96360), PpeSVP1 (XP_020422316), PpeSVP2 (XP_020409383), as listed by Falavigna et al.^106^, VvSVP (GSVIVT01001701001) and PtSVL (Potri007G010800.1). Full-length protein sequences and the maximum likelihood method were used to perform the phylogenetic analysis. FLC in Arabidopsis was set as the outgroup of the phylogenetic tree

Recent studies have shown that ABA regulates endodormancy, probably through MADS-box transcription factors that belong to the AGL24/SVP subfamily. In non-Rosaceae deciduous fruit trees, these MADS-box proteins are usually referred to as SVPs^63^, which are clustered in the same evolutionary branch as Arabidopsis SVPs (Fig. 4). The functions of these proteins during the endodormancy process have been verified using transgenic plants in several species, such as poplar and kiwifruit^19,20,63^. In poplar, SVP-like (SVL) acts as a central transcription factor integrating ABA signaling, ABA synthesis, GA synthesis, GA catabolism, and cell division, hence maintaining bud endodormancy^19,20,22^. In rosaceous plants, these dormancy-associated MADS-box proteins are named DAMs and are clustered separately from other SVPs. Six *DAM* genes, which are tandemly located at the end of chromosome 1 of *Prunus* species^64–66^, were first identified in the peach *evergrowing* mutant^64,67^. Ectopic expression of *PmDAM6* in poplar promoted the formation of dormant terminal buds and inhibited growth^65^. Zhao et al.^68^ found protein–protein interactions among DAM1–DAM6 proteins in *Prunus mume* and different expression patterns during bud endodormancy, indicating that DAMs might form different dimers during endodormancy. Wu et al.^69^ identified *DAM* and *SVP* genes in apple and found that overexpression of *MdDAMb* and *MdSVPa* caused delayed bud break. Ubi et al.^70^ and Saito et al.^71^ successively cloned three *DAM* genes (*PpyMADS13-1/-2/-3*) from Japanese pear ‘Kosui’ and showed that they are located on chromosomes 8 and 15. Afterwards, Liu et al.^72^ and Niu et al.^73^ also identified the same *DAM* genes from Chinese pear ‘Suli’ as those in Japanese pear and numbered them. Tuan et al.^54^ named *PpyMADS13-1 PpyDAM1*, corresponding to Niu’s *DAM3*. To better understand the evolution of *SVP* and *DAM* genes among different species, we unified the names of pear *DAM* genes according to the order of the chromosomes based on the new pear genome (unpublished data) and constructed a phylogenetic tree for SVP and DAM proteins in deciduous tree species (Fig. 4). *DAM* genes are *SVP-like* genes regulating the endodormancy process in Rosaceae, and they have not been found in grape, poplar, or Arabidopsis.

*DAM* genes are well studied in pear, peach, apple, and Japanese apricot. A molecular network associated with DAMs has already been demonstrated. Studies on pears have shown that low-temperature- and ABA-induced C-repeat binding factors (CBFs) directly bind to the promoters of *DAM* genes and activate their expression, promoting pear bud endodormancy^42,73,74^, while ABA-induced HD-ZIP protein HB22 also directly binds to the promoter of *DAM1* independently of CBF^52^. In Japanese apricot, CBFs not only bind to the promoter of *DAM6* but also form dimers with the DAM6 protein^75^. In the ABA signaling cascade, ABA-responsive AREB1 inhibits the expression of *DAM* genes^54^, while ABF3, an ABA-responsive transcription factor, induces *DAM3* expression, and ABF2 (a homologue to AREB1) disrupts activation by interacting with ABF3^40^. These interactions indicate the fine-tuned control of *DAM* gene expression, enabling plants to release endodormancy on time. In peach, the transcription factor TCP20 interacts with ABF2 and binds to the *DAM5*/*DAM6* promoter, inhibiting its expression^76^. In addition to transcription factors, miRNA6390 might be involved in the post-transcriptional regulation of *DAM* genes^73^, promoting the degradation of *DAM* transcripts. At the same time, DAM promotes the expression of *NCED*, which encodes a key enzyme in ABA synthesis; elevated *NCED* expression maintains a high ABA concentration in buds. Together, these roles of DAM form a negative feedback mechanism regulating pear bud endodormancy^54^. Furthermore, the expression of *DAM*s can negatively regulate the expression of *FT2*, a gene that promotes growth and flowering, during endodormancy^73^. In our recent study, overexpression of *DAM3* in pear calli resulted in downregulation of *Cyclin-D* and *EXPA1*, suggesting the potential regulation by DAMs of cell division and expansion^40^, which is similar to the role of SVL in poplar, indirectly inhibiting *Cyclin-D3* via EBB3^22^. However, other target genes of DAMs in deciduous fruit trees remain obscure.

**Fig. 5:**
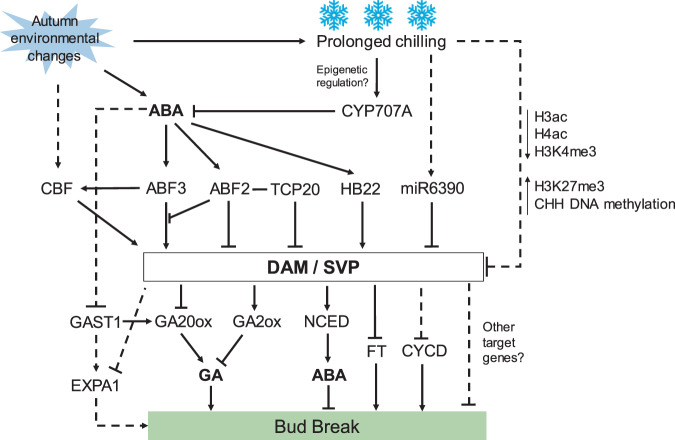
Model for DAM/SVP-centered molecular regulation of bud endodormancy in deciduous fruit trees. ABA is the primary hormone regulating DAM and SVP to maintain endodormancy. DAM and SVP proteins are transcription factors that integrate ABA signaling and GA biosynthesis and catabolism. Epigenetic regulation (such as H3ac, H3K4me3, H3K27me3, microRNAs, and DNA methylation) might be involved in the dormancy process during chilling exposure. CYP707A is a key enzyme in ABA catabolism, and the expression of *CYP707A* is upregulated with prolonged chilling. As *CYP707A* is regulated by epigenetic modification during seed dormancy and germination^86^, the possibility that *CYP707A* is similarly regulated by epigenetic modification during bud endodormancy needs further study. Arrowheads denote positive effects; blocked arrows denote negative effects. Dashed lines indicate indirect regulation or uncertain pathways

On the basis of previous studies in deciduous fruit trees and poplar, we summarized a DAM/SVP-centered endodormancy regulation network (Fig. 5). However, this molecular network cannot fully explain the mechanism of endodormancy release induced by chilling accumulation and the differences in the CR among different cultivars.

### Epigenetic regulation of bud endodormancy maintenance and release

Epigenetic regulation plays an important role in plant growth and development and in the response to environmental stresses. Epigenetic modification refers to reversible but heritable changes in gene expression and gene function without changes in the nucleotide sequence of the gene via mechanisms, including histone modification, DNA methylation, long noncoding RNA (lncRNA), and chromatin remodeling^77–79^. An increasing number of studies have shown that epigenetic modifications are deeply involved in the processes of both vernalization and seed dormancy, which are somewhat similar to bud endodormancy in the requirement for a certain period of chilling accumulation to finish vernalization or release seed dormancy. *FLOWERING LOCUS C* (*FLC*), which belongs to the *MADS-box* family, is an important gene regulating vernalization in Arabidopsis and has been proven to be regulated by epigenetic modification. Prolonged chilling during vernalization promotes the transcription of an antisense lncRNA named *COOLAIR* in the *FLC* locus and further alters histone modifications at the *FLC* gene locus through the polycomb-silencing complex pathway^80,81^, resulting in decreased H3K4me3 levels and increased H3K27me3 levels. These changes in histone methylation downregulate the expression of *FLC*, and the degree of downregulation was positively correlated with the duration of chilling accumulation^82,83^. Epigenetic modifications, including histone methylation and histone acetylation of genes involved in ABA and GA metabolism and signal transduction (e.g., *CYP707A*, *GA2ox*), also act in regulating seed dormancy release and germination^84–88^. In addition, DELAY OF GERMINATION1 (*DOG1*), involved in seed dormancy in ABA signaling^89^, is also regulated by H3K27me3 and H3K4me3 histone modifications during the seed dormancy cycle^90^. Therefore, endogenous hormone balance is critical for seed dormancy maintenance and release, and epigenetic regulation might activate and silence hormone-related genes.

Because the model of transcriptional regulation does not fully explain the mechanism of bud endodormancy release caused by subsequent chilling accumulation, some researchers have proposed that *DAM* genes are further regulated by epigenetic modifications similar to *FLC* during bud endodormancy. Santamaría et al.^91^ found differential expression of the histone modification-related genes *HUB2* and *GCN5L* in dormant and germinating buds in chestnut. Research in leafy spurge has shown that there was also a decrease in H3K4me3 and an increase in H3K27me3 in the *DAM1* promoter region during endodormancy release^92^, which is similar to the epigenetic modification of *FLC* during vernalization. Changes in H3K4me3, H3K27me3, and H3ac modifications of the promoter, coding region, and the second intron region of *DAM6* in two peach cultivars, were closely related to their specific chilling requirements and the date of endodormancy release^93^. During natural endodormancy, the reduction in H3K4me3 and H3ac modification of *AcSVP2*, which can delay bud break in kiwifruit, resulted in downregulation of *AcSVP2*, causing endodormancy release^94^. Recently, combined analysis of ChIP-seq with RNA-seq in sweet cherry revealed that the enrichment of H3K4me3 and H3K27me3 at target genes such as *PavDAM5* was positively and negatively correlated, respectively, with the expression of these genes during endodormancy^95^. In peach, downregulation of *DAM* genes was closely related to the increased level of H3K4me3 and CHH DNA methylation during chilling accumulation and during warmer conditions after chilling exposure, while noncoding RNAs also participated in regulating epigenetic modification^76,96^. However, in poplar, SVL seems not to be a target for H3K27me3 histone modification, but the expression of the downstream gene *EBB3* showed a negative correlation with H3K27me3 deposition after chilling exposure^19,22^. These studies indicate that *DAM/SVP* genes in deciduous fruit trees are potential target genes for epigenetic regulation during endodormancy maintenance and release, and that the changes in histone modification might be related to the chilling requirements of plants. Nevertheless, in the absence of mutants that alter chromatin regulation and affect endodormancy, most of these studies in deciduous fruit trees have yet to establish a causal link between chromatin regulation and endodormancy regulation.

Moreover, genes related to hormone metabolism might also be epigenetically regulated during bud endodormancy, similar to processes in seed dormancy. During seed dormancy and germination, *CYP707A*, which encodes an enzyme of ABA catabolism^86^, is most likely a target gene of epigenetic regulation. In pear, *CYP707A3* is upregulated with prolonged chilling accumulation during bud endodormancy^31,40^, suggesting that *CYP707A3* might similarly be a likely target of epigenetic regulation (Fig. 5). Song et al.^97^ reported that bZIP proteins are able to recruit the COMPASS-like complex to target genes and increase H3K4me3 modification to regulate unfolded protein and endoplasmic reticulum (ER) stress. Moreover, a study in peach demonstrated that bZIP proteins, together with ER stress and unfolded protein-related genes, are also involved in bud endodormancy during chilling accumulation^98^. Thus, further study will be needed to verify whether and how bZIP proteins are involved in epigenetic regulation during bud endodormancy processes.

In addition to histone modification, DNA methylation is also involved in the regulation of bud endodormancy^99–101^. Law and Sutte^102^ found that potato tubers showed high methylation levels during both endodormancy induction and release. Li et al.^103^ found that endodormant blueberry floral buds had higher methylation levels than those in the ecodormancy stage. Studies on apple showed that the changes in DNA methylation in buds during chilling accumulation and endodormancy release were significantly correlated with altered expression of some genes^104^. DNA methylation and small interfering RNA (siRNA) might be involved in the regulation of *MADS-box* genes in sweet cherry during the dormancy transition^100^. More recently, Rothkegel et al. demonstrated that DNA methylation might act as an early sensor of low temperature, leading to genotype-specific reprogramming and thus changing gene expression^105^.

In summary, epigenetic modifications such as histone modifications and DNA methylation are involved in bud endodormancy regulation, but how these modifications regulate endodormancy remains largely unknown, even in the model plant poplar. How the key genes related to bud endodormancy, such as *DAM* genes, are regulated by epigenetic modification still needs further study.

## Conclusions and perspectives

Floral bud endodormancy in deciduous fruit trees is an essential process that is complex and different from that in the model plant poplar. Nevertheless, the results of poplar endodormancy can provide a basis for research on fruit tree dormancy in the future. Low temperature and a short photoperiod are supposed to be the main environmental factors promoting bud endodormancy. In autumn, environmental changes cause the accumulation of ABA, leading to the differential expression of transcription factors (e.g., *ABF*, *CBF*, and *DAM*/*SVP*). DAM/SVPs in turn regulate the contents of phytohormones (ABA and GA) by regulating the expression of structural genes (e.g., *NCED*, *GA2OX,* and *GA20OX*) and forming a feedback regulation loop. At the same time, long-term exposure to winter low temperatures might cause epigenetic changes, which in turn result in changes in gene expression (e.g., *DAM* and *CYP7070A*), thus altering the contents of ABA and GA, leading to endodormancy release. However, there are still some major issues related to bud dormancy in fruit trees that need to be addressed in the future:

1. In some deciduous fruit trees, growth cessation occurs from early summer when the day length and temperature increase, but induction of endodormancy occurs in autumn when it is still warm in some regions. Although artificial low temperature and/or short-day length induce bud endodormancy, factors causing growth cessation and inducing endodormancy of deciduous fruit trees under natural conditions remain unclear.
2. Although DAMs/SVPs in some fruit trees, such as peach and kiwifruit, have been shown to promote endodormancy induction and maintenance, more genetic evidence is needed to verify the role of DAMs in bud endodormancy in other fruit trees. In addition, how DAM/SVP functions as a growth inhibitor remains largely unknown but might be clarified in the future by studying the target genes of DAMs/SVPs.
3. While much evidence has shown that epigenetic regulation participates in the dormancy cycle, how these epigenetic modifications (e.g., histone modification and DNA methylation) are enriched or reversed during dormancy processes remains unknown. It would be interesting to uncover how epigenetic marks are recruited to specific genes (e.g., *DAM* and *SVP*) and how those modifications are affected by chilling accumulation during bud endodormancy processes.
4. Chilling requirements are a complex quantitative trait. Although recent QTL analyses have provided useful information, the detailed genetic basis of CR traits remains obscure. The mechanisms underlying the formation of CR traits in floral buds need further study using newer genetic approaches, such as gene editing, genome-wide association studies, and genome-wide environmental association studies, which entail years of accumulated experience and materials.

Hormonal and transcriptional regulation can partially explain the dormancy cycle, while revealing the various epigenetic regulation mechanisms might explain the different CRs among different cultivars and under different environmental conditions. More genome-wide techniques, such as ChIP-seq and genome-wide association studies, should be used to reveal the genetic basis of the CR trait, while genetic transformation will allow us to verify the functions of key regulators of endodormancy.

## References

[1] Lang GA, Early JD, Darnell RL, Martin GC (1987). Endo-, para-, and eco-dormancy: physiological terminology and classification for dormancy research. Hort. science.

[2] Samish MR (1954). Dormancy in woody plants. Annu. Rev. Plant Physiol..

[3] Arora R, Rowland LJ, Tanino K (2003). Induction and release of bud dormancy in woody perennials: a science comes of age. Hort. Sci..

[4] Horvath DP, Anderson JV, Chao WS, Foley ME (2003). Knowing when to grow: signals regulating bud dormancy. Trends Plant Sci..

[5] Howe GT (1999). Physiological and genetic approaches to studying endodormancy-related traits in *Populus*. Hort. science.

[6] Singh RK, Svystun T, AlDahmash B, Jonsson AM, Bhalerao RP (2017). Photoperiod- and temperature-mediated control of phenology in trees - a molecular perspective. N. Phytol..

[7] Böhlenius H (2006). CO/FT regulatory module controls timing of flowering and seasonal growth cessation in trees. Science.

[8] Miskolczi P (2019). Long-range mobile signals mediate seasonal control of shoot growth. Proc. Natl Acad. Sci..

[9] Ding J (2018). GIGANTEA-like genes control seasonal growth cessation in Populus. N. Phytol..

[10] Ibáñez C (2010). Circadian clock components regulate entry and affect exit of seasonal dormancy as well as winter hardiness in *Populus* trees. Plant Physiol..

[11] Ramos-Sanchez JM (2019). LHY2 Integrates night-length information to determine timing of poplar photoperiodic growth. Curr. Biol..

[12] Azeez A, Miskolczi P, Tylewicz S, Bhalerao (2014). Rishikesh P. A tree ortholog of APETALA1 mediates photoperiodic control of seasonal growth. Curr. Biol..

[13] Karlberg A, Bako L, Bhalerao RP (2011). Short day-mediated cessation of growth requires the downregulation of AINTEGUMENTALIKE1 transcription factor in hybrid aspen. PLoS Genet..

[14] Tylewicz S (2015). Dual role of tree florigen activation complex component *FD* in photoperiodic growth control and adaptive response pathways. Proc. Natl Acad. Sci..

[15] Maurya JP (2020). Branching regulator BRC1 mediates photoperiodic control of seasonal growth in hybrid aspen. Curr. Biol..

[16] Ruttink T (2007). A molecular timetable for apical bud formation and dormancy induction in poplar. Plant Cell.

[17] Tylewicz S (2018). Photoperiodic control of seasonal growth is mediated by ABA acting on cell-cell communication. Science.

[18] Rinne PL (2011). Chilling of dormant buds hyperinduces *FLOWERING LOCUS T* and recruits GA-inducible 1,3-beta-glucanases to reopen signal conduits and release dormancy in Populus. Plant Cell.

[19] Singh RK (2018). A genetic network mediating the control of bud break in hybrid aspen. Nat. Commun..

[20] Singh RK, Miskolczi P, Maurya JP, Bhalerao RP (2019). A tree ortholog of SHORT VEGETATIVE PHASE floral repressor mediates photoperiodic control of bud dormancy. Curr. Biol..

[21] Zawaski C (2011). Repression of gibberellin biosynthesis or signaling produces striking alterations in poplar growth, morphology, and flowering. Planta.

[22] Azeez A (2021). EARLY BUD-BREAK 1 and EARLY BUD-BREAK 3 control resumption of poplar growth after winter dormancy. Nat. Commun..

[23] Conde D (2017). Overexpression of DEMETER, a DNA demethylase, promotes early apical bud maturation in poplar. Plant Cell Environ..

[24] Conde D (2017). Chilling-responsive DEMETER-LIKE DNA demethylase mediates in poplar bud break. Plant Cell Environ..

[25] Xin M (2019). Effect of latitude and altitude on flower bud differentiation of major apple cultivars. Acta Horticulturae Sin..

[26] Heide OM, Prestrud AK (2005). Low temperature, but not photoperiod, controls growth cessation and dormancy induction and release in apple and pear. Tree Physiol..

[27] Cooke JE, Eriksson ME, Junttila O (2012). The dynamic nature of bud dormancy in trees: environmental control and molecular mechanisms. Plant Cell Environ..

[28] Li S (2018). Protein changes in response to photoperiod during dormancy induction in peach leaves and flower buds. Sci. Hortic..

[29] George IS, Fennell AY, Haynes PA (2018). Shotgun proteomic analysis of photoperiod regulated dormancy induction in grapevine. J. Proteom..

[30] Zhang Q-j (2015). Identification of differentially expressed genes using digital gene expression profiles in Pyrus pyrifolia Nakai cv. Hosui bud release following early defoliation. Tree Genet. Genomes.

[31] Li J (2018). Abscisic acid (ABA) promotes the induction and maintenance of pear (*Pyrus pyrifolia* White Pear Group) flower bud endodormancy. Int. J. Mol. Sci..

[32] Li C, Junttila O, Heino P, Palva ET (2003). Different responses of northern and southern ecotypes of Betula pendula to exogenous ABA application. Tree Physiol..

[33] Wang H, G. D, Wang X, Li J (2006). Role of gibberellin and abscisic acid in peach bud endodormancy induction. J. Fruit. Sci..

[34] Or E, Belausov E, Popilevsky I, Bental Y (2000). Changes in endogenous ABA level in relation to the dormancy cycle in grapevines grown in a hot climate. J. Hortic. Sci. Biotechnol..

[35] Zheng C (2018). Abscisic acid catabolism enhances dormancy release of grapevine buds. Plant Cell Environ..

[36] Weinberger JH (1950). Chilling requirements of peach varieties. Proc. Am. Soc. Hortic. Sci..

[37] Richardson E, A S, S D, Walker DR (1974). A model for estimating the completion of rest for ‘Redhaven’ and ‘Elberta’ peach trees. Hort. science.

[38] Zhuang W (2012). Z. Advance on chilling requirement and its chilling models in deciduous fruit crops. J. Fruit. Sci..

[39] Feng L, M. C, Wu H, Qi K, Zhang S (2013). Comparative studies of chilling requirement and covering time of forcing cultivation of different pear cultivars in Nanjing. Chin. Agric. Sci. Bull..

[40] Yang Q (2020). ABA-responsive ABRE-BINDING FACTOR3 activates *DAM3* expression to promote bud dormancy in Asian pear. Plant Cell Environ..

[41] Liu, G. Study on molecular mechanism of pear dormancy. PhD thesis, Zhejiang University, (2013)..

[42] Li J (2019). PpCBFs selectively regulate *PpDAM*s and contribute to the pear bud endodormancy process. Plant Mol. Biol..

[43] Fan S (2010). Mapping quantitative trait loci associated with chilling requirement, heat requirement and bloom date in peach (*Prunus persica*). N. Phytol..

[44] Rowland LJ (2014). Construction of a genetic linkage map of an interspecific diploid blueberry population and identification of QTL for chilling requirement and cold hardiness. Mol. Breed..

[45] Castede S (2015). Mapping of candidate genes involved in bud dormancy and flowering time in sweet cherry (Prunus avium). PLoS ONE.

[46] Kitamura Y (2018). Identification of QTLs controlling chilling and heat requirements for dormancy release and bud break in Japanese apricot (Prunus mume). Tree Genet. Genomes.

[47] Gabay G (2017). Identification of QTLs associated with spring vegetative budbreak time after dormancy release in pear (*Pyrus communis* L.). Plant Breed..

[48] Gabay G (2018). High-resolution genetic linkage map of European pear (*Pyrus communis*) and QTL fine-mapping of vegetative budbreak time. BMC Plant Biol..

[49] Gabay G (2019). Transcriptome analysis and metabolic profiling reveal the key role of alpha-linolenic acid in dormancy regulation of European pear. J. Exp. Bot..

[50] Wang D (2015). Expression of ABA metabolism-related genes suggests similarities and differences between seed dormancy and bud dormancy of peach (*Prunus persica*). Front. Plant Sci..

[51] Zhuang W (2013). Comparative proteomic and transcriptomic approaches to address the active role of GA_4_ in Japanese apricot flower bud dormancy release. J. Exp. Bot..

[52] Yang Q (2018). PpHB22, a member of HD-Zip proteins, activates *PpDAM1* to regulate bud dormancy transition in ‘Suli’ pear (Pyrus pyrifolia White Pear Group). Plant Physiol. Biochem..

[53] Bai S (2013). Transcriptome analysis of Japanese pear (*Pyrus pyrifolia* Nakai) flower buds transitioning through endodormancy. Plant Cell Physiol..

[54] Tuan PA, Bai S, Saito T, Ito A, Moriguchi T (2017). Dormancy-associated MADS-Box (DAM) and the abscisic acid pathway regulate pear endodormancy through a feedback mechanism. Plant Cell Physiol..

[55] Zheng C (2015). Abscisic acid (ABA) regulates grape bud dormancy, and dormancy release stimuli may act through modification of ABA metabolism. J. Exp. Bot..

[56] Frisby JW, Seley SD (1993). Chilling of endodormant peach propagules: IV. Terminal shoot growth of cuttings, including gibberellic acid treatments. J. Am. Soc. Hortic. Sci..

[57] Zheng C (2018). Distinct functions for gibberellin during and after grapevine bud dormancy release. J. Exp. Bot.

[58] Yang Q (2019). PpyGAST1 is potentially involved in bud dormancy release by integrating the GA biosynthesis and ABA signaling in `Suli' pear (Pyrus pyrifolia White Pear Group). Environ. Exp. Bot..

[59] Karlberg A (2010). Analysis of global changes in gene expression during activity-dormancy cycle in hybrid aspen apex. Plant Biotechnol..

[60] Hao X (2019). Gene characterization and expression analysis reveal the importance of auxin signaling in bud dormancy regulation in tea plant. J. Plant Growth Regul..

[61] Chao WS, Doğramacı M, Horvath DP, Anderson JV, Foley ME (2017). Comparison of phytohormone levels and transcript profiles during seasonal dormancy transitions in underground adventitious buds of leafy spurge. Plant Mol. Biol..

[62] Shi Z (2018). Transient induction of a subset of ethylene biosynthesis genes is potentially involved in regulation of grapevine bud dormancy release. Plant Mol. Biol..

[63] Wu R (2017). Kiwifruit *SVP2* gene prevents premature budbreak during dormancy. J. Exp. Bot..

[64] Bielenberg DG (2008). Sequencing and annotation of the evergrowing locus in peach [*Prunus persica* (L.) Batsch] reveals a cluster of six MADS-box transcription factors as candidate genes for regulation of terminal bud formation. Tree Genet. Genomes.

[65] Sasaki R (2011). Functional and expressional analyses of PmDAM genes associated with endodormancy in Japanese apricot. Plant Physiol..

[66] Yamane H (2014). Regulation of bud dormancy and bud break in Japanese apricot (Prunus mume Siebold ^|^amp; Zucc.) and peach [Prunus persica (L.) Batsch]: a summary of recent studies. J. Jpn. Soc. Horticultural Sci..

[67] Li Z, Reighard GL, Abbott AG, Bielenberg DG (2009). Dormancy-associated MADS genes from the *EVG* locus of peach [*Prunus persica* (L.) Batsch] have distinct seasonal and photoperiodic expression patterns. J. Exp. Bot..

[68] Zhao K (2018). Comprehensive cloning of *Prunus mume* dormancy associated MADS-Box genes and their response in flower bud development and dormancy. Front. Plant Sci..

[69] Wu R (2017). SVP-like MADS box genes control dormancy and budbreak in apple. Front. Plant Sci..

[70] Ubi BE (2010). Molecular cloning of dormancy-associated MADS-box gene homologs and their characterization during seasonal endodormancy transitional phases of Japanese pear. J. Am. Soc. Hortic. Sci..

[71] Saito T (2013). Expression and genomic structure of the dormancy-associated MADS box genes MADS13 in Japanese pears (*Pyrus pyrifolia* Nakai) that differ in their chilling requirement for endodormancy release. Tree Physiol..

[72] Liu G (2012). Transcriptomic analysis of ‘Suli’ pear (*Pyrus pyrifolia* White Pear Group) buds during the dormancy by RNA-Seq. BMC Genomics.

[73] Niu Q (2016). Dormancy-associated MADS-box genes and microRNAs jointly control dormancy transition in pear (*Pyrus pyrifolia* White Pear Group) flower bud. J. Exp. Bot..

[74] Ahmad M (2019). Phylogenetic, molecular, and functional characterization of PpyCBF proteins in Asian pears (Pyrus pyrifolia). Int. J. Mol. Sci..

[75] Zhao K (2018). PmCBFs synthetically affect PmDAM6 by alternative promoter binding and protein complexes towards the dormancy of bud for *Prunus mume*. Sci. Rep..

[76] Wang Q (2020). Transcription factor TCP20 regulates peach bud endodormancy by inhibiting DAM5/DAM6 and interacting with ABF2. J. Exp. Bot..

[77] Chinnusamy V, Zhu JK (2009). Epigenetic regulation of stress responses in plants. Curr. Opin. Plant Biol..

[78] Law JA, Jacobsen SE (2009). Dynamic DNA methylation. Science.

[79] Zhao X (2018). Global identification of Arabidopsis lncRNAs reveals the regulation of MAF4 by a natural antisense RNA. Nat. Commun..

[80] Csorba T, Questa JI, Sun Q, Dean C (2014). Antisense *COOLAIR* mediates the coordinated switching of chromatin states at *FLC* during vernalization. Proc. Natl Acad. Sci. USA.

[81] Swiezewski S, Liu F, Magusin A, Dean C (2009). Cold-induced silencing by long antisense transcripts of an Arabidopsis polycomb target. Nature.

[82] Bastow R (2004). Vernalization requires epigenetic silencing of FLC by histone methylation. Nature.

[83] Sheldon CC, Rouse DT, Finnegan EJ, Peacock WJ, Dennis ES (2000). The molecular basis of vernalization: the central role of FLOWERING LOCUS C (FLC). Proc. Natl Acad. Sci. USA.

[84] Zheng J (2012). A novel role for histone methyltransferase KYP/SUVH4 in the control of Arabidopsis primary seed dormancy. N. Phytol..

[85] Wang Z (2013). Arabidopsis paired amphipathic helix proteins SNL1 and SNL2 redundantly regulate primary seed dormancy via abscisic acid-ethylene antagonism mediated by histone deacetylation. Plant Cell.

[86] Nonogaki H (2014). Seed dormancy and germination-emerging mechanisms and new hypotheses. Front. Plant Sci..

[87] Bouyer D (2011). Polycomb repressive complex 2 controls the embryo-to-seedling phase transition. PLoS Genet..

[88] Molitor AM, Bu Z, Yu Y, Shen W-H (2014). Arabidopsis AL PHD-PRC1 complexes promote seed germination through H3K4me3-to-H3K27me3 chromatin state switch in repression of seed developmental genes. PLoS Genet..

[89] Nee G (2017). DELAY OF GERMINATION1 requires PP2C phosphatases of the ABA signalling pathway to control seed dormancy. Nat. Commun..

[90] Footitt S, Muller K, Kermode AR, Finch-Savage WE (2015). Seed dormancy cycling in Arabidopsis: chromatin remodelling and regulation of DOG1 in response to seasonal environmental signals. Plant J..

[91] Santamaría ME, Rodríguez R, Cañal MJ, Toorop PE (2011). Transcriptome analysis of chestnut (Castanea sativa) tree buds suggests a putative role for epigenetic control of bud dormancy. Ann. Bot..

[92] Horvath DP, Sung S, Kim D, Chao W, Anderson J (2010). Characterization, expression and function of dormancy associated MADS-box genes from leafy spurge. Plant Mol. Biol..

[93] Leida C, Conesa A, Llácer G, Badenes ML, Ríos G (2012). Histone modifications and expression of *DAM6* gene in peach are modulated during bud dormancy release in a cultivar-dependent manner. N. Phytol..

[94] Wu R (2019). Histone modification and activation by SOC1-like and drought stress-related transcription factors may regulate AcSVP2 expression during kiwifruit winter dormancy. Plant Sci..

[95] Vimont, N. et al. ChIP-seq and RNA-seq for complex and low-abundance tree buds reveal chromatin and expression co-dynamics during sweet cherry bud dormancy. *Tree Genet. Genomes*10.1007/s11295-019-1395-9 (2019).

[96] Zhu H (2020). Thermal-responsive genetic and epigenetic regulation of DAM cluster controlling dormancy and chilling requirement in peach floral buds. Hortic. Res..

[97] Song ZT (2015). Transcription factor interaction with COMPASS-like complex regulates histone H3K4 trimethylation for specific gene expression in plants. Proc. Natl Acad. Sci. USA.

[98] Fu XL (2014). Roles of endoplasmic reticulum stress and unfolded protein response associated genes in seed stratification and bud endodormancy during chilling accumulation in *Prunus persica*. PLoS ONE.

[99] Prudencio AS (2018). DNA methylation analysis of dormancy release in almond (Prunus dulcis) flower buds using epi-genotyping by sequencing. Int. J. Mol. Sci..

[100] Rothkegel K (2017). DNA methylation and small interference RNAs participate in the regulation of MADS-box genes involved in dormancy in sweet cherry (*Prunus avium* L.). Tree Physiol..

[101] Santamaria ME (2009). Acetylated H4 histone and genomic DNA methylation patterns during bud set and bud burst in Castanea sativa. J. Plant Physiol..

[102] Law RD, Suttle JC (2003). Transient decreases in methylation at 5′-CCGG-3′ sequences in potato (Solanum tuberosum L.) meristem DNA during progression of tubers through dormancy precede the resumption of sprout growth. Plant Mol. Biol..

[103] Li B, Xia X, Liu S (2015). Changes in physiological and biochemical properties and variation in DNA methylation patterns during dormancy and dormancy release in blueberry (*Vaccinium corymbosum* L.). Plant Physiol. J..

[104] Kumar G, Rattan UK, Singh AK (2016). Chilling-mediated DNA methylation changes during dormancy and its release reveal the importance of epigenetic regulation during winter dormancy in apple (*Malus* x *domestica* Borkh.). PLoS ONE.

[105] Rothkegel K (2020). Dormant but active: chilling accumulation modulates the epigenome and transcriptome of prunus avium during bud dormancy. Front. Plant Sci..

[106] Falavigna, V. d. S., Guitton, B., Costes, E. & Andrés, F. I. Want to (Bud) Break Free: The Potential Role of DAM and SVP-Like Genes in Regulating Dormancy Cycle in Temperate Fruit Trees. *Front. Plant Sci*. 10.3389/fpls.2018.01990 (2019). 10.3389/fpls.2018.01990 PMC6335348 30687377

